# Characterization of TR‐107, a novel chemical activator of the human mitochondrial protease ClpP

**DOI:** 10.1002/prp2.993

**Published:** 2022-08-05

**Authors:** Emily M. J. Fennell, Lucas J. Aponte‐Collazo, Joshua D. Wynn, Kristina Drizyte‐Miller, Elisa Leung, Yoshimi Endo Greer, Paul R. Graves, Andrew A. Iwanowicz, Hani Ashamalla, Ekhson Holmuhamedov, Henk Lang, Donald S. Karanewsky, Channing J. Der, Walid A. Houry, Stanley Lipkowitz, Edwin J. Iwanowicz, Lee M. Graves

**Affiliations:** ^1^ Department of Pharmacology and the Lineberger Comprehensive Cancer Center University of North Carolina at Chapel Hill Chapel Hill North Carolina USA; ^2^ Department of Biochemistry University of Toronto Toronto Ontario Canada; ^3^ Women's Malignancies Branch Center for Cancer Research, National Cancer Institute, National Institutes of Health Bethesda Maryland USA; ^4^ Department of Radiation Oncology New York Presbyterian Brooklyn Methodist Hospital Brooklyn New York USA; ^5^ Madera Therapeutics LLC Chapel Hill North Carolina USA; ^6^ Institute of Theoretical and Experimental Biophysics Russian Academy of Sciences Pushchino Russian Federation; ^7^ Department of Chemistry University of Toronto Toronto Ontario Canada

**Keywords:** agonist, cell proliferation, mitochondria, oxidative phosphorylation, protease, small molecule, triple‐negative breast cancer

## Abstract

We recently described the identification of a new class of small‐molecule activators of the mitochondrial protease ClpP. These compounds synthesized by Madera Therapeutics showed increased potency of cancer growth inhibition over the related compound ONC201. In this study, we describe chemical optimization and characterization of the next generation of highly potent and selective small‐molecule ClpP activators (TR compounds) and demonstrate their efficacy against breast cancer models in vitro and in vivo. We selected one compound (TR‐107) with excellent potency, specificity, and drug‐like properties for further evaluation. TR‐107 showed ClpP‐dependent growth inhibition in the low nanomolar range that was equipotent to paclitaxel in triple‐negative breast cancer (TNBC) cell models. TR‐107 also reduced specific mitochondrial proteins, including OXPHOS and TCA cycle components, in a time‐, dose‐, and ClpP‐dependent manner. Seahorse XF analysis and glucose deprivation experiments confirmed the inactivation of OXPHOS and increased dependence on glycolysis following TR‐107 exposure. The pharmacokinetic properties of TR‐107 were compared with other known ClpP activators including ONC201 and ONC212. TR‐107 displayed excellent exposure and serum *t*_1/2_ after oral administration. Using human TNBC MDA‐MB‐231 xenografts, the antitumor response to TR‐107 was investigated. Oral administration of TR‐107 resulted in a reduction in tumor volume and extension of survival in the treated compared with vehicle control mice. ClpP activation in vivo was validated by immunoblotting for TFAM and other mitochondrial proteins. In summary, we describe the identification of highly potent new ClpP agonists with improved efficacy against TNBC, through targeted inactivation of OXPHOS and disruption of mitochondrial metabolism.

## Graphical abstract

Compounds tested for mitochondrial ClpP activation and binding, potent inhibition of breast cancer cell growth. TR107 selected and examined against multiple mitochondrial parameters. Efficacy of tumor regression by TR107 shown using MDA‐MB‐231 xenograft model.

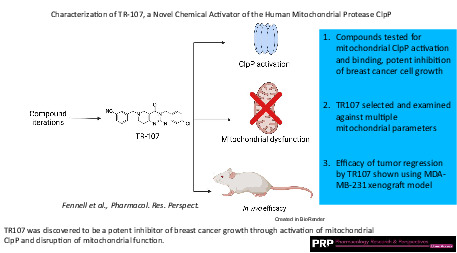

**Abbreviations**
ACO2: aconitase 2
ADEP: acyldepispeptide
AUC: area under the curve
BCL‐2: B‐cell lymphoma‐2
ClpP: mitochondrial matrix caseinolytic peptidase, proteolytic subunit
ClpX: mitochondrial matrix caseinolytic peptidase, chaperone subunit X
*C*_max_
: maximal plasma concentration
DMSO: dimethyl sulfoxide
DPBS: Dulbecco's phosphate‐buffered saline
DTT: dithiotheirtol
ECAR: extracellular acidification rate
ECL: enhanced chemiluminescence
F%: oral bioavailability
HSP60: heat shock protein 60
IC_50_
: inhibitory concentration 50
IDH1/2: isocitrate dehydrogenases 1/2
*K*_d_
: equilibrium dissociation constant
KO: knockout
OCR: oxygen consumption rate
OXPHOS: oxidative phosphorylation
PCR: polymerase chain reaction
PK: pharmacokinetic
POLRMT DNA: directed RNA polymerase, mitochondrial DNA
RIPA: radioimmunoprecipitation assay
SDHA: succinate dehydrogenase A
sgRNA: single guide RNA
SPR: surface plasmon resonance
*t*_1/2_
: elimination half‐life
TBST: Tris buffered saline‐Tween 20
TCA: tricarboxylic acid
TFAM: mitochondrial transcription factor A
TNBC: triple‐negative breast cancer
TUFM: mitochondrial elongation factor Tu
WT: wild‐type

## 1 INTRODUCTION

Triple‐negative breast cancer (TNBC) is the most aggressive breast cancer subtype and is associated with poor prognosis, shorter progression‐free, and overall survival compared with other breast cancer subtypes.^1^, ^2^, ^3^ Unlike other subtypes, targeted therapies (e.g., tamoxifen and trastuzumab) are ineffective against TNBC leaving patients with limited options of systemic chemotherapy, surgery, or radiation,^1^ or more recently immunotherapy.^4^, ^5^ As an alternative, considerable interest has developed in targeting mitochondrial metabolism as a potential approach to treat recalcitrant cancers.^6^, ^7^ Multiple studies now support the feasibility of targeting essential mitochondrial processes such as oxidative phosphorylation (OXPHOS), mitoribosomal activity, and TCA cycle function as a unique strategy to inhibit cancer cell proliferation.^8^, ^9^ This has led to the development of small‐molecule inhibitors of OXPHOS, TCA cycle enzymes (isocitrate dehydrogenases [IDH1/2], α‐ketoglutarate dehydrogenase), as well as modifiers of mitochondrial transcription (POLRMT) and lipid metabolism.^10^, ^11^ Included in this class of molecules are metformin and other clinical agents (BAY 87‐2243, IACS‐010759) designed to inhibit mitochondrial multimeric complex I enzyme that comprises the first step in the electron respiratory chain.^12^, ^13^, ^14^ While these have shown promising activities in preclinical studies, the clinical application of these agents has yet to be realized.^9^

The small‐molecule imipridone ONC201 was initially identified in a screen for TRAIL inducers and later shown to have antagonistic effects on the dopamine receptors D2/D3.^15^, ^16^ While the direct target was unresolved, multiple studies showed that ONC201 exhibited anticancer responses in diverse cancer models including breast, pancreatic, leukemia, and others^16^, ^17^, ^18^, ^19^, ^20^, ^21^, ^22^ leading to the clinical advancement of ONC201. The unexpected finding that ONC201 potently affected OXPHOS and mitochondrial metabolism in models of TNBC suggested an alternative mechanism of action.^23^ Importantly, the development of the chemically related TR compounds (Madera Therapeutics), including immobilizable analogs, facilitated the discovery of the mitochondrial matrix protease ClpP as the major target.^24^ Additional studies confirmed these findings and demonstrated that the activation of ClpP was essential for the anticancer properties of ONC201 and related analogs.^25^, ^26^

Modifiers of ClpP activity have previously shown anticancer properties,^27^, ^28^ with both inhibitors and activators of ClpP demonstrating antitumor efficacy for acute myelogenous leukemia.^29^, ^30^ Chemical activators of ClpP include diverse chemical structures such as the macrocyclic acyldepsipeptides (ADEPs) and D9 (Figure S1A). ADEP and D9 were both described in 2018 as human ClpP activating agents prior to the discovery of a similar mechanism of action for ONC201 and the TR compounds.^27^ Madera was the first to prepare a number of highly potent, cell‐permeable ClpP activators through modifications of peripheral functionality on the chemical core of ONC201, and later through changes to the chemical core.^24^ Detailed crystal structure analysis of ClpP and ONC201 further validated ClpP as a direct and specific binding partner.^25^

ClpP is a component of the ClpXP protein complex localized in the mitochondrial matrix. ClpP is a tetradecameric serine protease that forms a complex with hexameric AAA+ ClpX, an ATP‐dependent protein unfoldase that enables substrate recognition and unfolding prior to its degradation by ClpP.^30^, ^31^, ^32^, ^33^ The crystal structure of ClpXP has been solved from a number of species providing detailed insight into how this proteolytic protein complex is regulated. ClpX binds to a hydrophobic pocket in ClpP thereby facilitating the opening of the axial pore and passage of unfolded proteins into the central barrel of ClpP. Pharmacological activators of ClpP, such as the ADEPs, were the first small molecules shown to bind to this hydrophobic pocket and open the axial pore of ClpP in the absence of ClpX, allowing for nonspecific entry of proteins into the active site of ClpP.^28^, ^34^, ^35^

As part of the mitochondrial unfolded protein response, ClpXP canonically targets misfolded proteins to prevent the formation of protein aggregates in the mitochondria.^31^, ^36^ ClpXP also has regulatory roles in heme biosynthesis,^37^, ^38^, ^39^ mitophagy,^40^, ^41^ and reduction in reactive oxygen species levels following mitochondrial depolarization.^33^ ClpP is overexpressed in breast cancer,^42^ providing the potential for utilizing highly selective and potent ClpP activators as a novel approach to disrupt mitochondrial metabolic processes required for TNBC proliferation.

In this study, we present data on the characterization of a novel class of highly potent and selective ClpP activators. We focused on one compound, TR‐107, and show that it induces the downregulation of selected mitochondrial proteins, impairs OXPHOS, and inhibits TNBC growth in a ClpP‐dependent manner, with markedly improved potency compared with the imipridones (ONC201, 206, 212). Analysis of the pharmacokinetic properties of TR‐107 demonstrated high systemic drug levels following oral administration. Furthermore, the efficacy of TR‐107 in mouse xenograft models of MDA‐MB‐231 TNBC cells showed that TR‐107 was well tolerated and inhibited tumor growth and increased animal survival in a manner consistent with ClpP activation. In summary, these studies identify TR107 as one of the most potent and selective ClpP activators with anticancer potential.

## 2 MATERIALS AND METHODS

### 2.1 Cell culture

Human TNBC cell line SUM159 was a generous gift from Dr. Gary Johnson (the University of North Carolina at Chapel Hill). MDA‐MB‐231 (WT and CLPP‐KO) cells were provided by Dr. Stanley Lipkowitz (National Cancer Institute). MDA‐MB‐231 cells were cultured in RPMI‐1640 media (Gibco, 11875‐093) supplemented with 10% fetal bovine serum (FBS; VWR‐Seradigm, 97068‐085), and 1% antibiotic–antimycotic (anti/anti; ThermoFisher Scientific, 15240062). SUM159 cells were cultured in Dulbecco's modified Eagle's medium: Nutrient Mixture F‐12 (DMEM/F12; Gibco, 11330‐032) supplemented with 5% FBS, 5 μg/ml insulin (Gibco, 12585014), 1 μg/ml hydrocortisone, and 1% anti/anti. Cells were maintained at 5% CO_2_ and 37°C and periodically tested for mycoplasma.

### 2.2 CRISPRi cell lines

sgRNA against human ClpP (Eurofins Genomics) was annealed and ligated into vector VDB783. This vector was transformed into DH5α cells and the presence of sgRNA was confirmed by colony PCR. Lentivirus was produced in HEK293T cells following transfection with plasmid and jetPRIME (PolyPlus) as previously described.^43^ Clonal populations of SUM159 cells infected with dCas9‐KRAB lentivirus were clonally isolated to ensure dCas9‐KRAB expression. Isolated SUM159 cells were infected with ClpP sgRNA lentivirus followed by additional selection passage in growth media supplemented with 2.5 mg/ml puromycin.

### 2.3 Synthesis of compounds

All TR compounds were prepared by Madera Therapeutics, LLC as previously described in the patent literature.^44^ ONC201, ONC206, and ONC212/TR‐31 were purchased from Selleck Chemicals, LLC (S7963, S6853, S8673, respectively).

### 2.4 Viability assays

Total cell counting assays were performed by seeding MDA‐MB‐231 cells (WT and ClpP‐KO; 4000 cells/well) or SUM159 (WT and ClpP‐KO; 1000 cells/well) in a 96‐well plate (Perkin Elmer, 6005050) and allowed to adhere overnight. The growth medium was then aspirated and replaced with 100 μl of growth medium supplemented with the drug at concentrations indicated in the figure or figure legends. Cells treated with vehicle control (0.1% DMSO, Sigma‐Aldrich D2650) were used as a negative control in all experiments, and cells were incubated with drug‐containing media for 24, 48, or 72 h as indicated in figure legends. Media was then aspirated and replaced with 100 μl Dulbecco's phosphate‐buffered saline (DPBS Gibco, 14190‐144) containing 1 μg/ml Hoechst stain (ThermoFischer Scientific, H3570) and allowed to incubate at 37°C for 30 min. The total cell number was then quantified using the Celigo Imaging Cytometer (Nexcelom).

### 2.5 Immunoblotting

MDA‐MB‐231 or SUM159 cells were plated (100 000 cells/well) on a six‐well Costar plate (Corning, 3516) and incubated with compounds as described above for cell viability assays. Following treatment, cells were rinsed three times with 2 ml of cold DPBS and lysed using RIPA buffer (20 mM Tris [pH 7.4], 137 mM NaCl, 10% glycerol, 1% Nonidet P‐40, 0.5% deoxycholate, 2 mM EDTA) supplemented with 2 mM Na_3_VO_4_, 10 mM NaF, 0.0125 μM calyculin A, and complete protease inhibitor cocktail (Roche Diagnostics, 11873580001). Cell lysates were clarified and immunoblotted as described earlier.^45^ Membranes were incubated with primary antibodies (Appendix S1) diluted 1:1000 in 1% fish gelatin (Sigma‐Aldrich, G7041)/TBST overnight at 4°C, removed, washed 3 × 5 min in TBST, and placed in their respective 2° antibodies (1:10 000 dilution in 5% milk/TBST) for 1 h. Membranes were washed 3 × 5 min in TBST then incubated in ECL reagent (BioRad, 170‐5061) and imaged using a Chemidoc MP (BioRad) or Odyssey Fc (LI‐COR). Acquired images were processed/quantified using Image Lab software (BioRad) or Image Studio Lite (LI‐COR).

### 2.6 Caspase activity assay

Caspase‐3/7 activity was analyzed using a fluorescent peptide substrate (Ac‐DEVD‐AMC, Cayman Chemical, 14986) as previously described.^45^ Briefly, cells were plated at 1 × 10^5^ cells per well (6 cm^2^ plate, Corning, 430166) and treated for 24 h with 10 μM ONC201, 150 nM TR‐57, 100 nM TR‐107 (10X IC_50_) as well as 0.1% DMSO or 100 nM staurosporine. The samples were collected in lysis buffer (50 mM HEPES [pH: 7.4], 5 mM CHAPS, and 5 mM DTT), and caspase activity was measured by monitoring fluorescence (ex/em: 360/460 nm) using a SpectraMax i3x (Molecular Devices).

### 2.7 ClpP activity assay

Measurement of in vitro activity of purified recombinant human ClpP was performed with a slight modification to the method previously described.^46^ Briefly, ClpP activity was measured through the degradation of casein‐FITC (Sigma‐Aldrich, C0528) in the presence of indicated compounds. The activity of ClpP proteolytic subunit (41 μg/ml [1.4 μM]) was measured in assay buffer (20 mM HEPES [pH 8.0], 100 mM KCl, 1 mM DTT, and 100 μM ATP) using 235 μg/ml fluorogenic casein‐FITC substrate. Enzyme and compounds were mixed and incubated in assay buffer for 30 min before adding casein‐FITC substrate. The kinetics of substrate degradation fluorescence was monitored in 96‐well plates (PerkinElmer, 8059‐21401), and fluorescence was recorded at 490/525 nm (ex/em) using SpectraMax i3x (Molecular Devices). Linear slope of fluorescence signal was used to measure ClpP activity and normalized to DMSO control, expressed as RFU/h.

### 2.8 Surface plasmon resonance

For the surface plasmon resonance (SPR) experiments, recombinant human ClpP was purified as previously described.^46^ SPR measurements were performed on a Biacore X100 instrument (Cytiva Life Sciences) at 25°C in buffer (25 mM sodium phosphate, pH 7.5, 200 mM KCl, 0.05% Tween‐20, and 0.004% DMSO). ClpP was immobilized onto flowcell two of a CM5 chip (Cytive Life Sciences) using the amine coupling wizard using Biacore X100 Control Software. A pulse of a mixture of NHS and EDC activated the CM5 chip surface. ClpP was diluted to 100 μg/ml in 10 mM sodium acetate, pH 4.5 immediately prior to use and injected over the activated surface for 180 s. The remaining activated sites were blocked with a pulse of ethanolamine. 12 000 RU of ClpP was captured using this procedure. Flowcell 1 was unmodified and served as a reference. For TR‐107 binding to ClpP, a concentration series of TR‐107 in DMSO was diluted into the buffer to achieve 0.004% final DMSO concentration in all samples. Three replicates were injected for each concentration. Data were blank subtracted and analysis was performed in Biacore X100 Evaluation Software (Cytiva Life Sciences). The steady‐state response was calculated 15 s before the end of injection with a window of 15 s. Data were fitted to a one‐site Langmuir binding model.

### 2.9 Mitochondrial respiration analysis

Cellular oxygen consumption and extracellular acidification rates (OCR and ECAR, respectively) were measured using Seahorse XFe96 (Agilent). MDA‐MB‐231 cells were plated (15 000 cells/well) in Seahorse XF96 plates (Agilent, 102416‐100) and allowed to adhere overnight. Cells were then treated with 25 or 50 nM TR‐107 for 24 h. On the day of Seahorse XF analysis, the growth medium was replaced with an XF base medium (Agilent, 103575‐100) supplemented with appropriate concentrations of TR‐107. After measurement of basal OCR/ECAR, 1 μM Oligomycin, 1 μM FCCP, and 1 μM Rotenone/Antimycin A (Agilent, 103015‐100) were added at indicated time points. OCR/ECAR were measured every 6.5 min for 73 min.

### 2.10 Mitochondrial DNA (mtDNA) copy number analysis

Mitochondrial DNA copy number was determined as previously described.^23^ Briefly, genomic DNA was isolated from tissue lysate with DNeasy Blood & Tissue kit (Qiagen, 69504). mtDNA copy number was determined by quantitative PCR with a human mitochondrial to nuclear DNA ratio kit (Takara Bio USA, 7246).

### 2.11 Pharmacokinetic analysis

The compounds were evaluated for pharmacokinetic properties in ICR mice (Sino‐British SIPPR/BK Lab Animal Ltd) by Shanghai Medicilon, Inc. The compounds were administered either intravenously (tail vein) or orally (oral gavage) in vehicle (5% DMSO, 10% Solutol [Kolliphor HS 15, Sigma‐Aldrich, 42966] in sterile DPBS, 85% water) to three male mice per study arm. Blood was taken via the orbital venous plexus (0.03 ml/timepoint) at 0.083, 0.25, 0.5, 1, 2, 4, 8, and 24 h unless otherwise noted. Samples were placed in tubes containing heparin sodium and stored on ice until centrifuged. Blood samples were centrifuged at 6800*g* for 6 min at 2–8°C within 1 h following sample collection and stored at −80°C. Proteins from plasma sample aliquots (20 μl) were precipitated by the addition of MeOH (400 μl) containing an internal standard (tolbutamide,100 ng/ml), vortexed for 1 min, and centrifuged at 18 000*g* for 7 min. The supernatant (200 μl) was transferred to 96 well plates for analysis.

Analysis of samples was conducted as follows. An aliquot of supernatant (1 μl) was analyzed for the parent compound by LC‐MS/MS using a Luna Omega C18 column (2.1 × 50 mm, 1.6 μm; Phenomenex, 00B‐4747‐B0) with 0.1% formic acid/water and 0.1% formic acid/acetonitrile gradient system, and a TQ6500+ triple quad mass spectrometer (positive ionization mode). In the case of TR‐107, the parent compound and internal standard (IS) were detected with electrospray ionization in positive mode (ESI+) using multiple reaction monitoring (MRM) of mass transition pairs at *m/z* of 391.2/247.2 (TR‐107) and 271.1/172.0 (IS, tolbutamide) amu. The calibration curve was obtained by spiking known concentrations (2 to 2000 ng/ml) of TR‐107 into blank mouse plasma. The PK parameters including Area Under the Curve (AUC_[0–∞]_) elimination half‐life (*t*_1/2_), maximal plasma concentration (*C*_max_), and oral bioavailability (*F*%) were analyzed by noncompartmental methods.

### 2.12 Protein‐binding studies to plasma proteins

Protein‐binding studies were performed by Eurofins Panlabs, Inc. This assay utilizes equilibrium dialysis in a microplate format, as previously described.^47^ Briefly, this analysis is used to determine the bound and unbound fraction of the drug and calculate the percentage of the test compound binding to murine plasma proteins.

### 2.13 Mouse xenograft studies

#### 2.13.1 Mice

Female athymic nude mice (Crl:NU[Ncr]‐Foxn1^*nu*^, Charles River) were 8 weeks old with body weight (BW) range of 19.0–27.0 g on Day 1 of the study. Animals were fed ad libitum water (reverse osmosis, 1 ppm Cl) and NIH 31 Modified and Irradiated Lab Diet® consisting of 18.0% crude protein, 5.0% crude fat, and 5.0% crude fiber. Mice were housed on irradiated Enrich‐o'cobs™ Laboratory Animal Bedding in static microisolators on a 14‐h light cycle at 20–22°C and 40%–60% humidity. Charles River Discovery Services North Carolina (CR Discovery Services) specifically complies with the recommendations of the Guide for Care and Use of Laboratory Animals with respect to restraint, husbandry, surgical procedures, feed and fluid regulation, and veterinary care. The animal care and use program at CR Discovery Services is accredited by the Association for Assessment and Accreditation of Laboratory Animal Care International.

### 2.14 Tumor cell culture

MDA‐MB‐231 human breast adenocarcinoma cells were grown to mid‐log phase in RPMI‐1640 supplemented with 10% FBS, 2 mM glutamine, 100 units/ml sodium penicillin G, 25 μg/ml gentamicin, 100 μg/ml streptomycin sulfate, 0.075% sodium bicarbonate, 10 mM HEPES, and 1 mM sodium pyruvate. Cells were cultures at 37°C and 5% CO_2_.

### 2.15 In vivo implantation and tumor measurement

MDA‐MB‐231 cells were harvested during exponential growth and resuspended in cold, sterile DPBS. Each animal received an orthotopic injection of 5 × 10^6^ cells (0.05 ml volume) into the mammary fat pad. Tumor growth was monitored as the average tumor size approached the target range of 60–100 mm^3^. Tumors were measured in two dimensions via caliper and volume was calculated using the following formula:

$$\text{Tumor Volume}=\frac{w^{2}\times l}{2},$$

where *w* is the width and *l* is the length (mm) of the tumor. Tumor weight was estimated based on the assumption that 1 mg = 1 mm^3^. Fourteen days later (Day 1), animals were sorted into groups (*n* = 10 per group) with a mean tumor volume of 70–73 mm^3^.

### 2.16 Treatment

Mice began dosing on Day 1 as summarized in Table S1. Compounds were administered orally (p.o.) in a dosing volume of 10 ml/kg. Twice daily doses (B.I.D) were administered at least 6 h apart.

### 2.17 Endpoint and tumor growth delay analysis

Tumors were measured using calipers biweekly and animals were euthanized upon reaching tumor volume > 1500 mm^3^ or on the final study day (Day 36), whichever came first. Time to endpoint (TTE) for analysis was calculated for animals that exited the study due to tumor volume using the following equation:

$$\mathrm{TTE}=\frac{\log_{10}\left(\text{endpoint volume}\right)-b}{m},$$

where TTE is expressed in days, endpoint volume is expressed in mm^3^, *b* is the *y*‐intercept, and *m* is the slope of the line obtained by linear regression of a log‐transformed tumor growth data set. This data set consisted of the first observation that exceeded 1500 mm^3^ and the preceding three consecutive measurements. Animals that did not reach the tumor volume threshold were assigned a TTE value of 36 days (equivalent to study end). In instance where this equation yields a TTE day preceding the day prior to reaching the endpoint or exceeding the day of reaching the endpoint, a linear interpolation was performed to approximate TTE. Animals documented as having died of nontreatment‐related causes due to accident or unknown etiology were excluded from TTE calculations and all further analysis. Animals documented having died due to treatment‐related deaths or nontreatment‐related due to metastasis were assigned a TTE value equivalent to the day of death. Treatment outcome was evaluated from tumor growth delay (TGD) defined as the increase in the median TTE in the treatment group compared with the control group.

### 2.18 Sampling

On Day 36, all animals in all groups were sampled. Tumors were excised, divided into two parts, and weighed. Part 1 was preserved in a 5 ml fixative solution (4% formaldehyde, 2% glutaraldehyde in 0.1 M cacodylate buffer) and stored at 4°C. Part 2 was trimmed to be less than 0.5 cm in at least one dimension, submerged in five volumes of RNAlater solution, and stored at −20°C.

### 2.19 Statistical analysis

Statistical analysis for viability, mitochondrial respiration, ClpP activity assays, and animal studies was performed using Prism 9 (GraphPad). Analysis of pharmacokinetic data was performed using FDA certified pharmacokinetic program Phoenix WinNonlin 7.0 (Pharsight). In the mouse xenograft study, study groups experiencing toxicity beyond acceptable limits (>20% group mean BW loss or >10% treatment‐related deaths) or having fewer than five evaluable observations were not included in the statistical analysis. Survival was analyzed by Kaplan–Meier method, and the logrank (Mantel‐Cox) test was used to determine the significance of overall survival experiences based on TTE values.

## 3 RESULTS

### 3.1 New TR chemical scaffolds are highly potent inhibitors of TNBC growth

**FIGURE 1:**
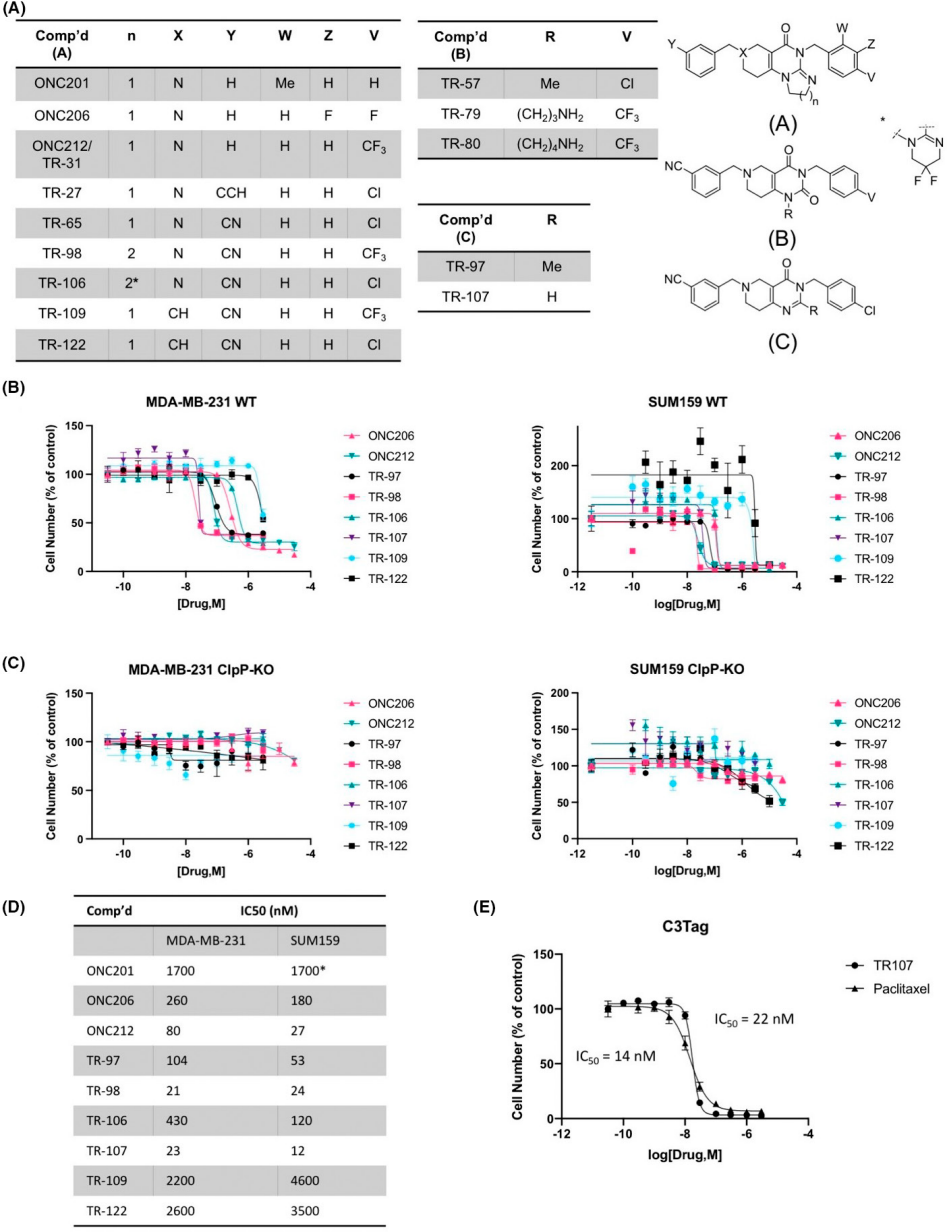
New TR compound analogs potently inhibit breast cancer cell growth in a ClpP‐dependent manner. (A) Comparison of novel TR compound chemical structures to ONC201 and analogs. Cell viability assays of (B) Wildtype (WT) and (C) ClpP knockout (ClpP‐KO) MDA‐MB‐231 and SUM159 cells. Cells were treated with indicated drug concentrations for 72 h and imaged following addition of Hoechst stain as described in Section 2. Values represent mean ± SEM normalized to DMSO control, representative of *N* = 3. (D) IC50 values in WT cells for compounds represented in (B). Values represent mean IC50 value (nM), *N* = 3. *ONC201 IC50 in MDA‐MB‐231 obtained from data shown in Figure 4D, SUM159 cells previously published.^48^ (E) Cell viability assay of C3Tag cells following TR‐107 and paclitaxel treatment. Cells were treated with drug concentrations as indicated for 72 h and imaged following Hoechst stain addition. Representative of *N* = 2. Average IC50 values indicated to the left and right of the graph for paclitaxel and TR‐107, respectively.

Madera Therapeutics synthesized multiple small molecules containing modifications to the multiring core system using the optimized peripheral functionality from TR‐65 and similar analogs. This included modification of the core imipridone structure with the appropriate addition of nitrile, halide, and trifluoromethyl groups to the two benzyl moieties. The structures of these compounds and other established ClpP agonists (i.e., ADEPS and D9) are shown in Figure 1A, Figure S1A, or a previous publication.^24^

We tested the TR compounds' ability to inhibit the growth of two commonly studied TNBC cell lines. Many of these new compounds showed significantly enhanced potency of cell growth inhibition in the MDA‐MB‐231 and SUM159 cell models compared with ONC201 and ONC206. Of these, TR‐98 and TR‐107 demonstrated the greatest growth inhibitory potency, comparable to the previously reported TR‐57 and TR‐65.^24^ For example, TR‐107 inhibited cell growth with an IC_50_ of 12 nM and 23 nM in SUM159 and MDA‐MB‐231 cells, respectively (Figure 1B). By contrast, TR‐109 and TR‐122, devoid of a key nitrogen atom of the six‐membered three‐piperideine system (X in structure A, Figure 1A), were less potent, suggesting the importance of a reported hydrogen bond interaction with tyrosine 118 of ClpP.^25^ This hydrogen bond is one of the three hydrogen bonds observed in the human ClpP–ONC201 complex structure, involving glutamate 82 and glutamine 107 (through a bridging water molecule) that anchor the molecule to the hydrophobic site of ClpP.^25^ Taken together, our data with TR‐97, TR‐98, and TR‐107 show that significant chemical modification of the imipridone core is tolerated and that the imidazoline moiety of the imipridone core is not required for high potency.

To confirm the ClpP dependence of the new TR compounds, the growth inhibitory effects of these compounds were compared in matched pairs of wild‐type (WT) and ClpP‐KO MDA‐MB‐231 and SUM159 cell lines. Compared with the dose‐dependent inhibition observed in the WT cells, no significant growth inhibition was observed in the ClpP–KO cells, providing evidence that ClpP is the major target for these molecules (Figure 1C). To compare the response to TR‐107 in another model of TNBC, we examined the effects of TR‐107 on C3(1)‐Tag cells and compared the potency of growth inhibition to the tubulin inhibitor paclitaxel, an established therapeutic. The C3(1)‐Tag model is a transgenic murine model of TNBC that shares many of the essential characteristics of human TNBC.^49^ TR‐107 potently inhibited C3(1)‐Tag cell proliferation with an IC_50_ value (22 nM), similar to paclitaxel (14 nM) (Figure 1D), which was also similar in potency to that observed with the MD‐MBA‐231 and SUM159 cells (23 and 12 nM, respectively).

### 3.2 Characterization of novel activators of ClpP

**FIGURE 2:**
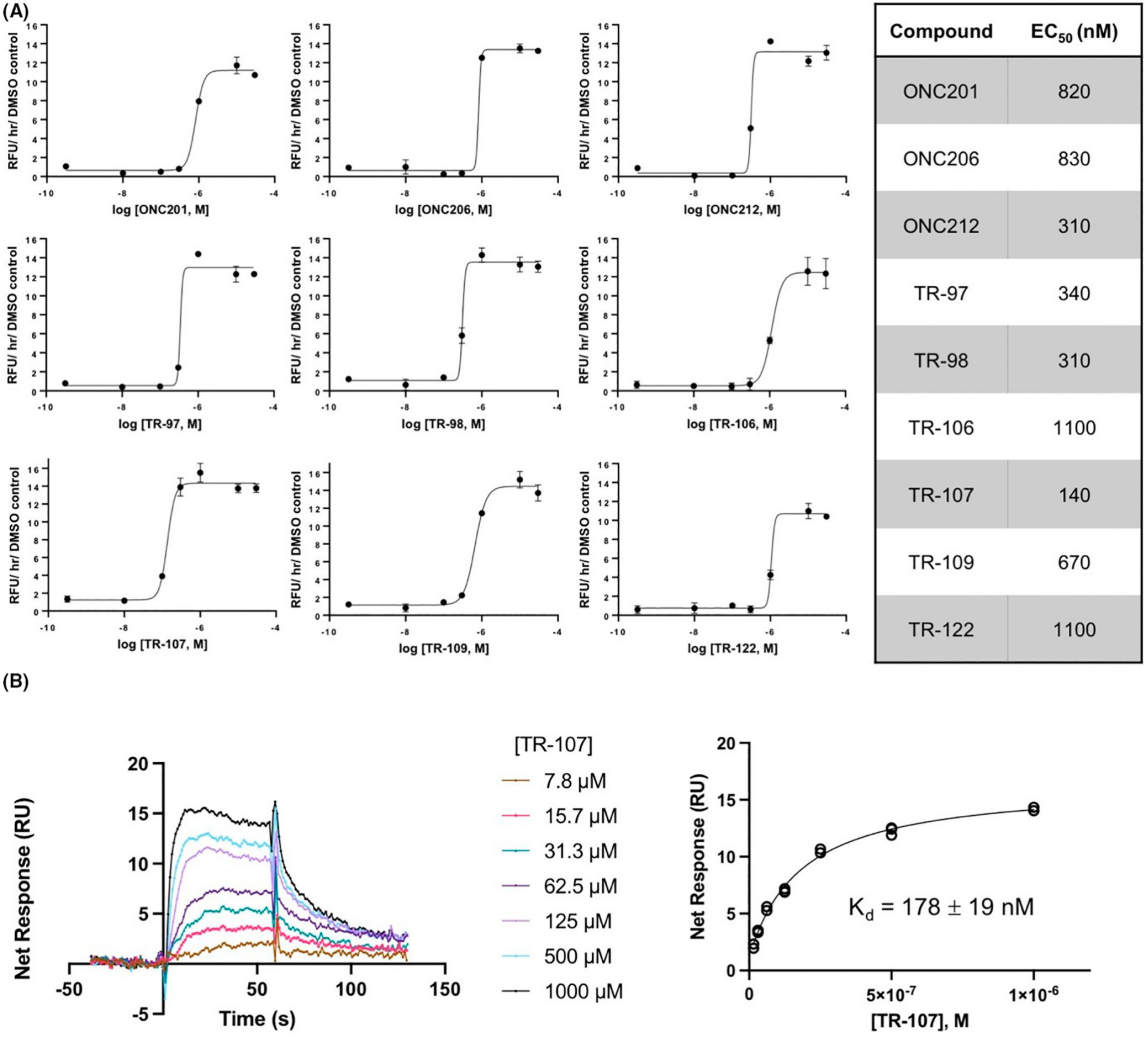
New TR compounds are potent activators of mitochondrial ClpP. (A) In vitro ClpP activity assays was performed as described in Section 2 using fluorescent casein‐FITC. Increase in fluorescent intensity relative to DMSO controls is shown; values represent mean ± SEM for technical replicates. EC50 values are presented in the table to the right. (B) Left panel—surface plasmon resonance (SPR) sensorgrams of TR‐107 binding to recombinant human ClpP coupled on the chip. Right panel—binding curves are shown as response units (RU) versus ligand concentration at steady state and fitted to a one‐site Langmuir binding model. The apparent *K*_*d*_ value obtained from the fit is shown.

We examined the ability of the new TR compounds to bind and activate ClpP. Select TR compounds were incubated with purified recombinant human ClpP and ClpP proteolytic activity was determined using the in vitro activity assay described in Section 2. All compounds showed dose‐dependent increases in ClpP activity with the greatest potency of ClpP activation observed with TR‐107, followed by TR‐98 and TR‐97 (EC_50_ = 140, 310, and 340 nM, respectively). Consistent with reduced effects on cell growth, TR‐109 and TR‐122 were also weaker activators of ClpP in vitro (Figure 2A). Direct binding of TR‐107 to purified ClpP was also examined. Surface plasmon resonance (SPR) measurement of TR‐107 binding to ClpP revealed values equivalent to that observed for ClpP activation (*K*_*d*_ ~180 nM) (Figure 2B). Higher concentrations of the compound were required for ClpP activation or binding in vitro, compared with cell growth inhibition. However, compounds that potently activate ClpP in vitro also potently inhibited cell growth as observed above (Figure 1) and as previously reported.^24^

### 3.3 TR‐107 inhibits TNBC growth without a significant increase in apoptosis

Since TR‐107 showed excellent potency in these assays, this compound was selected for further testing on cell growth inhibition and apoptosis. MDA‐MB‐231 or SUM159 cells were incubated with TR‐107 and total cell counts were determined every 24 h for up to 72 h following treatment at the indicated concentrations. As shown in Figure S1B, TR‐107 potently (at concentrations >10 nM) inhibited the increase in cell number compared with vehicle control in both cell lines. By comparison, cell growth was not inhibited in the ClpP‐KO cells with TR‐107 concentrations as high as 1 μM (Figure S1B). Because the highest concentration of TR‐107 tested in MDA‐MB‐231 or SUM159 cells did not reduce the total cell number below the initial seeded number, we tested for evidence of apoptosis.

Caspase‐3/7 assays were performed on lysates from the MDA‐MB‐231 or SUM159 cells following 24‐h compound treatment at indicated concentrations. Caspase activity was quantified as described in Section 2. Staurosporine (STS)‐treated cells showed a strong increase in caspase‐3/7 activity as expected, whereas no increase in caspase‐3/7 activity was detected in lysates from ONC201‐, TR‐57‐, or TR‐107‐treated cells (Figure S1C). Similarly, no increase in PARP cleavage after ONC201, TR‐57, or TR‐107 treatment was observed (data not shown). Taken together, these findings indicate that the TR compounds show growth inhibitory effects without substantially increasing apoptosis, consistent with previously reported results for ONC201 in MDA‐MB‐231 cells.^23^

### 3.4 TR‐107 induces time‐ and dose‐dependent reduction of multiple mitochondrial proteins

**FIGURE 3:**
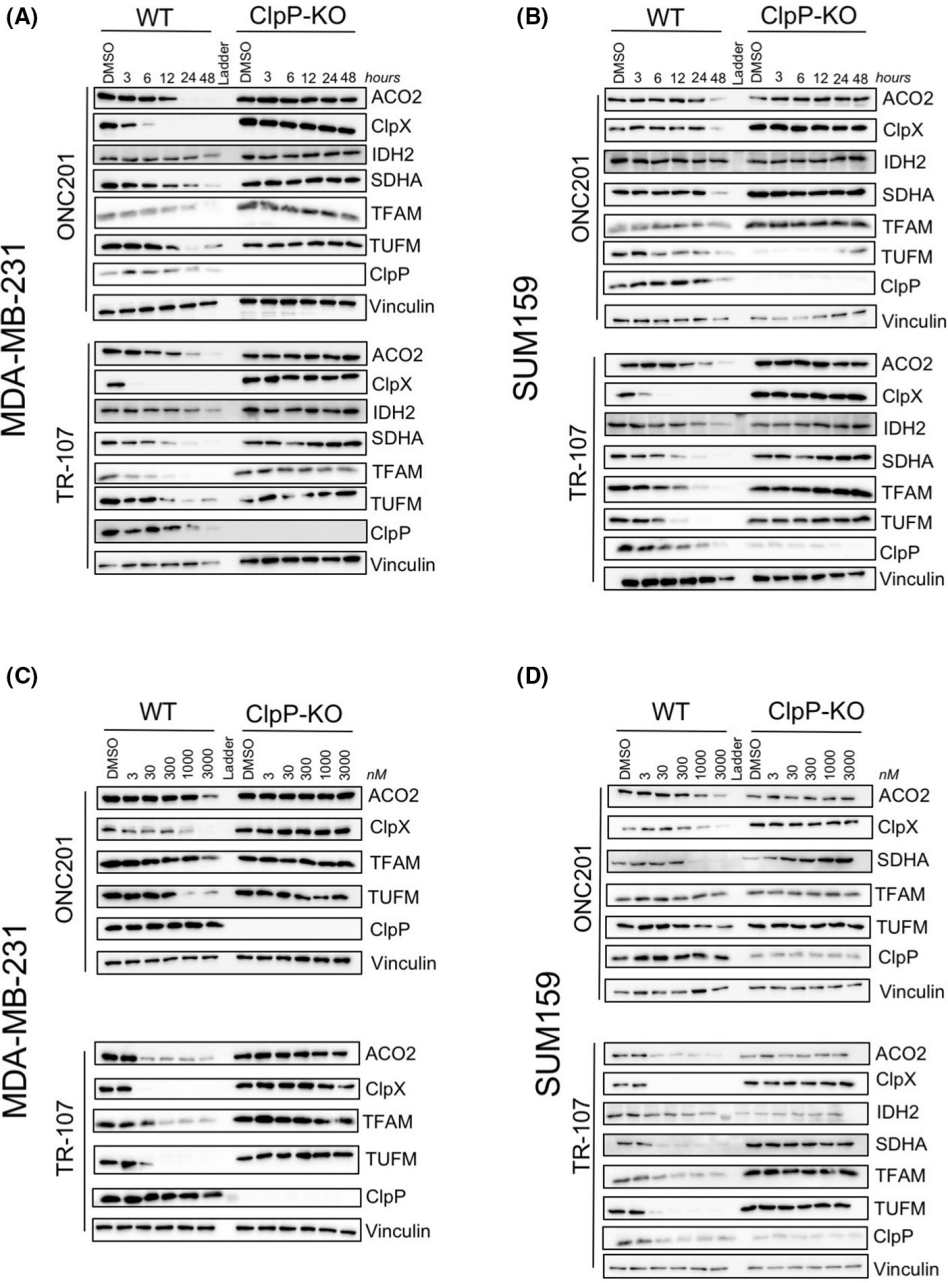
TR‐107 induces loss of mitochondrial proteins in TNBC cells in a ClpP‐ dependent manner. Triple‐negative breast cancer cells MDA‐MB‐231 (A, C) and SUM159 (B, D) were treated with 10 mM ONC201 or 100 nM TR‐107 for indicated time points (A, B) or indicated doses (C, D) of ONC201 or TR‐107 for 24 h and immunoblot was performed for various mitochondrial metabolic proteins. Representative of *N* = 3.

We and others reported that treatment of MDA‐MB‐231 or SUM159 cells with ONC201 or TR‐57 resulted in the reduction of the mitochondrial proteins elongation factor Tu (TUFM) and transcription factor A (TFAM).^23^, ^24^ Since ClpP is a mitochondrial matrix protease, we compared the time‐ and dose‐dependent effects of TR‐107, TR‐57, and ONC201 on additional mitochondrial proteins in MDA‐MB‐231 and SUM159 cells. As determined by immunoblotting, both TUFM and TFAM protein levels began to decline ~6 h after treatment with 100 nM TR‐107, 150 nM TR‐57, or 10 μM ONC201 and showed a complete or near‐complete loss by 24 h (Figure 3A,B; Figure S2A). We also observed the time‐ and dose‐dependent loss of aconitase (ACO2) and isocitrate dehydrogenase (IDH2), two key TCA cycle enzymes beginning at ~12–24 h following treatment (Figure 3A–D; Figure S2A,B). Succinate dehydrogenase A (SDHA) and complex I subunit NDUFS3, both OXPHOS proteins, were observed to decline by 12–24 h (Figure 3A,B; Figure S2A–C). ClpX, the ATPase subunit of the ClpXP complex, was previously observed to decline after 24 h of ONC212 treatment in pancreatic cancer models.^50^ We observed ClpX decline rapidly (~3 h) after TR compound addition (Figure 3A,B; Figure S2A). The loss of all these proteins was dose‐dependent and occurred at concentrations at or above the respective growth inhibitory (IC_50_) value for each compound with TR‐107 showing the most potent effects (Figure 3C,D; Figure S2B). By comparison, the levels of these proteins were not affected in the ClpP‐KO cell lines, even at the highest concentration of the compound tested (Figure 3, Figure S2).

Heat shock protein 60 (HSP60) is a mitochondrial matrix chaperone required for maintaining the folding of imported proteins and HSP60 inhibitors are being investigated as potential anticancer compounds.^51^ Immunoblotting for HSP60 protein after TR‐107 treatment of MDA‐MB‐231 cells showed that HSP60 levels were significantly reduced in a time‐dependent manner (Figure S2D). DNA‐directed RNA polymerase, mitochondrial (POLRMT) is a TFAM interacting protein involved in the replication of mitochondrial DNA. Recent studies have shown the efficacy of inhibiting POLRMT as a potential anticancer approach.^10^ Because of the observed decline in TFAM, we investigated whether POLRMT was similarly affected by these treatments. Immunoblotting data showed that POLRMT followed a dose‐dependent decline in full‐length protein level following TR compound treatment (Figure S2E). Together, these results demonstrate significant effects of TR‐107 on mitochondrial proteins involved in energetic and homeostatic processes in a time‐, dose‐ and ClpP‐dependent manner.

### 3.5 TR‐107 inhibits OXPHOS in MDA‐MB‐231 cells

**FIGURE 4:**
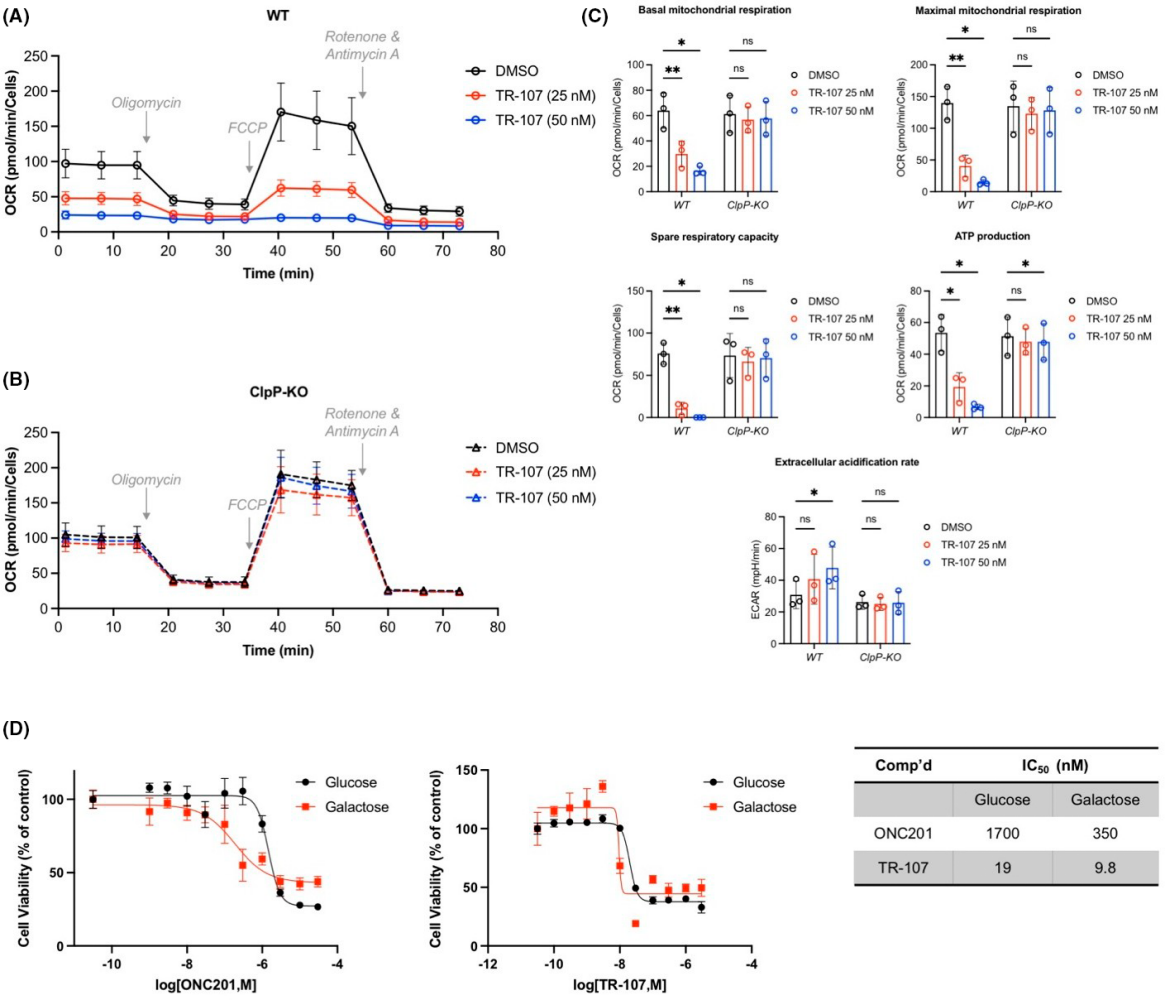
TR‐107 reduces mitochondrial metabolic functions in MDA‐MB‐231 cells. Oxygen consumption rate (OCR) of MDA‐MB‐231 cells treated with DMSO or TR‐107 at indicated concentrations for 24 h was measured by Seahorse XF Analyzer as described in Section 2. Trace of oxygen consumption data following TR‐107 treatment in (A) WT and (B) ClpP‐KO MDA‐MB‐231 cells, representative of *N* = 3 experiments. Indicated compounds were added at concentrations described in Section 2. (C) Bar charts of data shown in (A) and (B), values represent mean ± SEM, *N* = 3. *p*‐value <.05 (*), .001 (**). (D) Cell viability assays (Hoechst stain, 72 h) of MDA‐MB‐231 cells treated with ONC201 (left) or TR‐107 (right) at indicated concentrations in media containing either glucose (black) or galactose (red). Values represent mean ± SEM, representative of *N* = 3. Average IC50 values are presented in table (right).

Because previous studies showed inhibition of OXPHOS by ONC201^23^, ^25^ and ADEP,^52^ we examined the effects of TR‐107 on mitochondrial oxygen consumption and respiration using a Seahorse XF Analyzer. Incubation of MDA‐MB‐231 cells with TR‐107 (25, 50 nM) resulted in a dose‐dependent decline in oxygen consumption rate (OCR) (Figure 4A). By contrast, this was not observed in ClpP‐KO MDA‐MB‐231 cells (Figure 4B). We observed a significant dose‐dependent decline in basal mitochondrial respiration, ATP production‐linked oxygen consumption, maximal respiration, and spare respiratory capacity following TR‐107 treatment in WT but not ClpP‐KO cells (Figure 4C). Correspondingly, TR107 incubation caused a dose‐dependent increase in extracellular acidification (ECAR, Figure 4C). Taken together, our results demonstrate substantial disruption of OXPHOS by TR107 in a dose‐ and ClpP‐dependent manner.

As shown by Greer et al., ONC201 treatment resulted in a greater dependence on glycolysis for cell survival, and switching media carbon sources from glucose to galactose enhanced cell growth inhibition by ONC201.^23^ We similarly compared the effects of incubating cells in galactose instead of glucose on cell growth inhibition by TR‐107. As expected for ONC201, galactose shifted the dose–response curve for growth inhibition by ~5‐fold (IC_50_ = 1.7 μM [glucose] and 350 nM [galactose]). While the effects on TR‐107 on growth inhibition after galactose substitution were much less marked (~2‐fold change; IC_50_ = 19 nM [glucose] and 9.8 nM [galactose]) (Figure 4D), these results are consistent with OXPHOS inactivation and an increased reliance on glycolysis after TR‐107 treatment.

### 3.6 Pharmacokinetic properties of TR‐107

With TR‐107 and other leading TR compounds showing significantly enhanced potency over ONC201 and functional effects indicative of specific ClpP activation, we compared the PK properties of a set of TR compounds, ONC201 and ONC212/TR‐31. The PK studies were conducted by tail vein injection or oral gavage in ICR mice as described in Section 2. Based on the enhanced potency of this set of TR compounds, we anticipated an effective oral dose to be ~10 mg/kg or less in a murine model of breast cancer. Therefore, we chose to evaluate the oral exposure of these agents with a single dose of 10 mg/kg and in many cases a 2 mg/kg i.v dose allowing for an F% determination. Importantly, the results of our studies showed significant differences in PK properties among these compounds (Table 1).

**TABLE 1:** Pharmacokinetic analysis of select compounds in mice

Comp'd	Admin (mg/kg)	*T*_1/2_ (h)	*C*_max_ (mg/ml), [μM]	AUC (0–∞; h*ng/ml)	*F*%
ONC201	2.0, i.v.	0.26	122 [0.31]	50.7	N/A
	10, oral	0.31	8.99 [0.023]	3.13 (1X)	1.2
	25, oral	—	195 [0.50]	145 (46X)	N/A
TR‐27	2.0, i.v.	1.06	154 [0.35]	155	N/A
	10, oral	1.16	127 [0.29]	255	33
ONC212/TR‐31	2.0, i.v.	1.68	950 [2.2]	638	N/A
	10, oral	1.49	282 [0.64]	449	14
TR‐65	2.0, i.v.	0.73	636 [1.5]	351	N/A
	10, oral	0.95	294 [0.67]	584	33
TR‐57	2.0, i.v.	1.52	1240 [3.0]	886	N/A
	10, oral	1.40	1710 [4.1]	2710	61
TR‐107	10, oral	0.90	1440 [3.7]	2360	N/A

*Note*: Each arm of pharmacokinetic were performed as described in Section 2. Blood collection for ONC201 at 25 mg/kg (oral) was 0.083–4 h. The TR compounds were administered intravenously (“i.v.”) (2.0 mg/kg) or by oral gavage (“oral”) (10 mg/kg) in vehicle, described in Section 2. *A 2.5× increase in oral gavage dose gave a 46× increase in exposure (AUC). Three male ICR mice were utilized per study arm.

Of the impridone analogs closely related to ONC201, TR‐27, ONC212/TR‐31, and TR‐65 showed enhanced exposure over ONC201, increased F%, and half‐lives ranging from ~0.95 to 1.68 h. Our initial studies of ONC201 using either an intravenous dose of 2 mg/kg or an oral dose of 10 mg/kg showed markedly different results than what has been previously reported for higher doses. Both arms of our study showed a short *t*_1/2_ (0.26–0.31 h) and poor oral bioavailability (*F*% = 1.2%) versus a reported *t*_1/2_ of 6.4 h in mice.^18^ Notably, a 25 mg/kg oral dose of ONC201 showed a 46X increase in exposure (AUC) vs the 10 mg/kg study. In addition, ONC212/TR‐31 displayed a terminal half‐life of 1.49 h (10 mg/kg), whereas the reported *t*_1/2_ of an oral dose of 125 mg/kg was 4.3 h^53^ (Table 1).

TR‐57 and TR‐107 displayed the highest exposure (AUC) of the tested agents when administered at 10 mg/kg orally (2710 and 2360 hr ng/ml respectively, Table 1). Both compounds showed rapid absorption via oral administration with an *F*% of 61% (TR‐57). In addition, protein‐binding studies for TR‐107 show a serum‐free fraction of 10% (mouse, Figure S3C). Because of the favorable PK characteristics observed with TR‐107 and its efficient molecular design, this compound was selected for advancement in animal xenograft studies described below.

### 3.7 TR‐107 inhibits tumor growth in an MD‐MBA‐231 mouse xenograft model

To determine the in vivo antitumor efficacy of TR‐107, we examined dose‐dependent responses using an MDA‐MB‐231 mouse xenograft model. MDA‐MB‐231 cells in Matrigel were orthotopically injected into the flank mammary fat pad of female NCr nu/nu mice (10/group) as described in Section 2 and Appendix S1. Vehicle control (Group 1) and TR‐107 treatments (4 mg/kg [Group 2], 8 mg/kg [Group 3]) were administered by oral gavage at frequencies described in Table S1.

**FIGURE 5:**
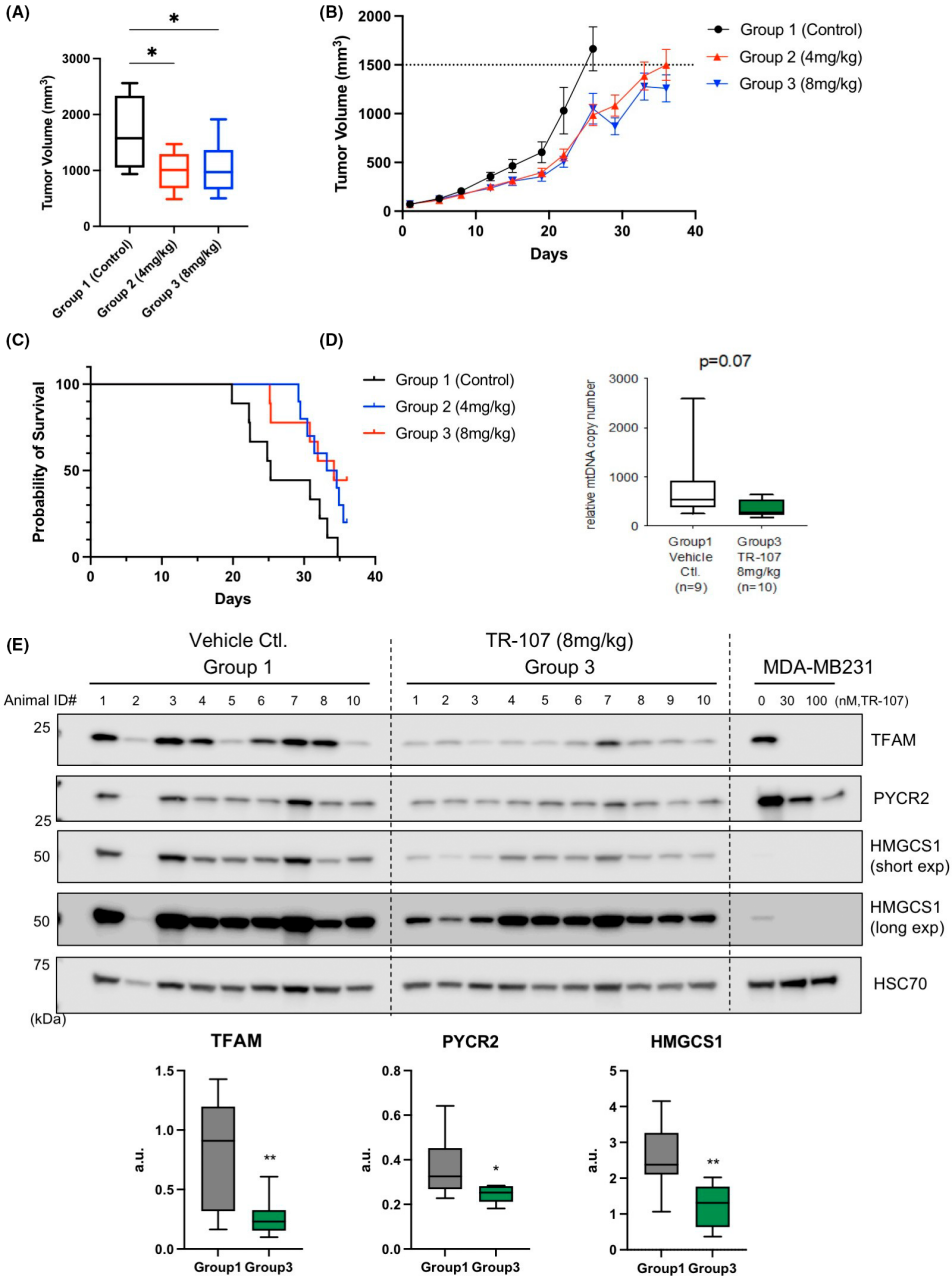
TR‐107 prevents tumor growth in MDA‐MB‐231 xenograft model. (A) Box‐ and‐whisker plot of tumor volume (mm^3^) of MDA‐MB‐231 xenografts following TR‐107 treatment at indicated concentrations at Day 26 (Table S2). *N* = 8 (Group 1) and 10 (Groups 2, 3). (B) Average tumor volume following TR‐107 administration as described in Methods. The tumor volume threshold (1500 mm^3^) is indicated by dashed line. *N* = 10 per group. Values represent mean ± SEM. (C) Kaplan‐Meier graph of mouse survival following TR‐107 administration as described in Methods and Table S2. Long‐rank (Mantel‐Cox) *p*‐value of 0.0163. Individual mice were recorded as leaving the study following death, tumor volume exceeding threshold (1500 mm^3^), or end of study period. (D) Box‐and‐whisker plot of relative mitochondrial DNA (mtDNA) copy number present in mouse xenograft lysate following conclusion of mouse study. *N* = 9 (Group 1) and *N* = 10 (Group 3). (E) Immunoblot (top) from tumor lysates of mouse xenografts following administration of vehicle control or 8 mg/kg TR‐107. Each animal ID represented an individual mouse. Two images are shown for HMGCS1 representing a short exposure (“short exp”) and long exposure (“long exp”). Quantification of these blots is shown in the box‐and‐whisker plot (bottom) with indicated *p*‐values (<.05 (*) and .01 (**)).

The results of these studies demonstrated a dose‐ and time‐dependent reduction in tumor volume. The mean tumor volume decreased for both treatment groups (G2, G3) with a ~50% reduction in tumor volume observed on day 26 (Figure 5A). Comparing the two different groups over the course of the study did not reveal significant advantages of one dosing regimen over the other in tumor volume reduction (Figure 5A,B) or survival (Figure 5C). A ~35% increase in median survival was also observed in mice treated with TR‐107 for both groups (Figure 5C). Consistent with ClpP activation, analysis of the tumor lysates from control and treated animals (G3), showed a substantial loss of mitochondrial DNA (Figure 5D). TR‐107 treatment was well tolerated and less than 5% weight loss was observed even at the highest dosing regimen (Figure S3). Effects of ONC201 and TR compounds on ClpP activation in vivo were further interrogated through immunoblot of xenograft tumor lysates (Figure 5E). These analyses demonstrated a significant loss of TFAM consistent with our cell culture studies. Additionally, mitochondrial pyrroline‐5‐carboxylate reductase 2 (PYCR2) and 3‐hydroxy‐3‐methylglutaryl‐CoA synthase 1 (HMGCS1) protein levels were significantly reduced in these tumor lysates. Taken together, TR‐107 showed significant efficacy in the prevention of tumor growth, consistent with ClpP activation, in the MDA‐MB‐231 mouse xenograft model.

## 4 DISCUSSION

ONC201, a screening hit with previously described CNS activity,^54^ was the first imipridone molecule shown to be effective against a variety of cancer models.^17^, ^20^, ^50^, ^54^, ^55^, ^56^, ^57^ Chemically similar derivatives of ONC201 (ONC206 and ONC212/TR‐31) were recently identified and both ONC201 and ONC206 are under evaluation in clinical trials. Despite evidence suggesting that ONC201 acts through effects on the dopamine receptor,^55^ the discovery of the mitochondrial protease ClpP as the target for ONC201 and chemically related compounds^24^, ^25^, ^26^ has redefined our understanding of the anticancer mechanism of these agents. Moreover, the identification of ClpP as the major target has enabled the design and characterization of highly potent and specific ClpP activators. In this study, we describe the advancement of the chemically diverse TR compounds as selective ClpP activators, which includes molecules of different chemical scaffolds from which agents for preclinical assessment were selected. We characterize one of these compounds, TR‐107, for effects on mitochondrial metabolism and protein turnover, PK properties, and TNBC growth inhibition both in vitro and in vivo. Importantly, these studies further demonstrate the pivotal role of ClpP activation in the mechanism of TR‐107 action.

From the collection of TR compounds, we were able to perform initial structure–activity relationship (SAR) studies using cell growth inhibition and ClpP activation as determinants. Nanjing Gator Meditech was the first to accurately determine that for the imipridone scaffold, substitution at the V‐ and Z‐positions by small lipophilic moieties was preferential to substitution at the W‐position, yielding nM activity in cancer cells (R.^58^). Our survey of residues for the other benzyl ring determined that the nitrile residue at position Y was preferred. The combination of these two optimized benzyl residues on the imipridone scaffold (TR‐65) resulted in markedly improved potency with regards to both ClpP activation and growth inhibition^24^ compared with ONC201 and ONC206.

Two key chemical features of the imipridone chemical core are basic nitrogen of the 3‐piperidine moiety and an imbedded acylguanidine. The importance of the basic nitrogen of the piperideine residue to activity is demonstrated through the substitution of the nitrogen atom with the carbon atom (e.g., X position, TR‐109, TR‐122, Figure 1A). Both TR‐109 and TR‐122 are significantly less potent than TR‐65 displaying μM IC_50_ values for TNBC cells. We previously reported the modification of the acylguanidine moiety to prepare TR‐57 and the homologated analogs TR‐79 and TR‐80 for target identification studies.^24^ We now report that further modification of TR‐57 removing the carbonyl oxygen and methyl residue (R group) gives TR‐107, a compound of equal potency. In addition, expansion of the five‐membered ring of the imipridone scaffold by one methylene unit yields TR‐98, a compound that is equipotent to TR‐65. In summary, the additional modifications described here resulted in two of the most potent ClpP activators (TR‐98 and TR‐107) so far reported and further support that the imipridone chemical scaffold is not a requirement of potent ClpP activation.

Because of its high potency in cell growth and ClpP activation assays, TR‐107 was selected for further mechanistic, pharmacokinetic, and animal model studies. Other attributes include a low molecular weight (<400 amu), highly potent, and defined ClpP dependence, excellent PK properties, and efficacy against TNBC models both in vivo and in vitro. Using two independent ClpP–KO cell lines, our studies confirm the mitochondrial protease ClpP as the key target for TR‐107 and related compounds. This includes effects on cell growth, OCR, and mitochondrial protein level as determined by immunoblotting. While ONC201, at concentrations far higher than that is necessary to activate ClpP or inhibit cell growth, has shown effects on dopamine receptors D2/D3,^55^, ^59^ our data^24^ and that of others^23^, ^25^ strongly argues against a major role of the dopamine receptors in growth inhibition.

In strong support of a mitochondrial mechanism of action, we observed TR‐107 treatment results in reduction of multiple mitochondrial proteins. Some of the most significantly affected proteins, in addition to TFAM and TUFM, included ACO2 and IDH2, which catalyze essential reactions in the TCA cycle. Similarly, respiratory chain proteins NDUFS3 and SDHA declined strongly. Last, we observed the ClpP‐dependent loss of other essential mitochondrial proteins including POLRMT and HSP60, both of which are predicted to have significant effects on mitochondrial function through regulation of mitochondrial gene replication and protein chaperone functions. Both POLRMT and HSP60 have been shown to be viable drug targets for cancer research,^10^, ^51^ and provide potential further explanations for the anticancer properties of these compounds. While this is a partial list of all mitochondrial proteins observed to decline following TR‐107 treatment, based on these results, we propose that the inactivation of key mitochondrial functions is, in part, responsible for the growth inhibition observed in these studies.

Mitochondrial dysfunction and metabolic reprogramming are accepted hallmarks of cancer.^7^, ^48^, ^60^, ^61^, ^62^ Inhibiting mitochondrial processes such as OXPHOS has gained interest as an alternative approach to combatting cancer.^13^, ^63^, ^64^, ^65^, ^66^, ^67^, ^68^ Our studies confirm the original findings of Greer et al. that ONC201 targets mitochondrial metabolism. In addition to TCA cycle enzymes, we observed proteins with essential roles in OXPHOS that were reduced after ONC201 and TR‐107 exposure. The inhibition of OXPHOS was confirmed by mitochondrial respiration analysis and galactose experiments. Interestingly, substituting media glucose for galactose had a much greater effect on the IC_50_ values of ONC201 compared to TR‐107. These data further illustrated the superior potency of TR‐107 and suggest that the effects of TR‐107 may be less dependent on nutritional conditions (e.g., glucose availability) compared with ONC201. Taken together, the results of our studies demonstrate an increased reliance on glycolysis as a compensatory response to inhibition of mitochondrial metabolism by ClpP activation.

Previous attempts to inhibit OXPHOS or specific enzymes in the TCA cycle have met with limited success for cancer treatment. The repurposing of metformin [1,1‐dimethylbiguanide] as an anticancer agent has been investigated in both research and clinical settings.^63^, ^67^, ^69^ As a weak inhibitor of mitochondrial complex I, metformin appears to lack the potency and specificity to effectively treat cancer as a single agent or in combination via complex I inhibition. The complex I inhibitor, BAY 87‐2243, is a highly potent inhibitor of complex I with a more complex chemical structure than metformin featuring lipophilic pharmacophores absent in metformin. Notably, clinical evaluation of BAY 87‐2243 was terminated after treatment of patients (NCT01297530). A focused contemporary approach by Stuani et al. to target mutant forms of IDH 1 and 2 and thereby reducing the oncometabolite (R)‐2‐hydroxyglutarate (2‐HG), led to agents that showed initial efficacy but lost their effectiveness with established disease.^70^

As single agents, we observed that ONC201 and the TR compounds induced a growth stasis response in these TNBC models. Even at the highest concentrations tested, little evidence for apoptosis was observed. In this way, our results are consistent with earlier studies in TNBC that showed a similar absence of apoptosis after treatment with ONC201.^21^, ^23^ Recent studies suggest that the addition of TRAIL or TRAIL receptor agonists induces an apoptotic response in cells that have previously displayed a growth stasis response to ONC201.^71^, ^72^, ^73^ Moreover, a combination of imipridones with BCL‐2 inhibitors has shown promise for increased cancer cell apoptosis.^19^, ^74^ Given the greatly improved potency of the TR compounds, it will be important to determine if low concentrations of TR compounds more effectively sensitize cells to TRAIL treatment or synergize with TRAIL ligand or other agents to increase apoptosis.^75^

Last, the development of potent and selective ClpP agonists addresses a key limitation for ONC201, ONC206, and ONC212/TR‐31 series of compounds regarding the requirement of high doses (50–130 mg/kg) to achieve in vivo efficacy.^53^ The enhanced potency of ONC206 and TR‐31/ONC212 in cellular growth assays did not result in lower in vivo efficacious doses. The pharmacokinetic data reported herein, coupled with previously reported results, points to a complex picture of the pharmacokinetic behavior of ONC201 and ONC212/TR‐31 at therapeutically relevant doses.

TR‐107 at an oral dose of 10 mg/kg results in similar *t*_1/2_ and serum exposure (AUC) to an equivalent oral dose of TR‐57. In addition to improved PK properties, TR‐107 effectively reduced tumor volume and increased median survival in vivo at 4 and 8 mg/kg dosages. Further analysis of xenografts revealed a decrease in TFAM protein levels and mitochondrial DNA following TR‐107 administration. Similar results were observed with additional mitochondrial proteins (PYCR2), confirming ClpP activation in vivo and providing additional evidence for the mitochondrial mechanism of action.

In summary, our studies show chemical optimization of ONC201 has resulted in novel ClpP activators with increased potency in both in vitro and in vivo studies. TR‐107 showed excellent potency in TNBC cell growth inhibition and provide further evidence that ClpP activation inhibits growth through loss of mitochondrial metabolic functions. Our in vivo studies show that these compounds are highly orally bioavailable, well tolerated, and effective at reducing tumor burden in an MDA‐MB‐231 murine xenograft model. In total, our observations support the clinical evaluation of TR‐107 for the treatment of TNBC.

### 4.1 Limitations

There are a number of limitations in our studies that should be mentioned. The characterization of the direct effects of these compounds on purified recombinant ClpP may not accurately replicate the ClpP activation kinetics in vivo. This may be a factor of the concentration of ClpP, the artificial substrates or the assay conditions used that underestimate the potency of ClpP activation by these compounds. In fact, our data from the cell‐based analysis of dose‐dependent ClpP activation, very closely parallels the effects observed on growth inhibition, for both ONC201 and the TR compounds. Therefore, we believe that the cell‐based assays are a better indicator of ClpP activation efficacy and further reflect potential differences in compound cell permeability as well. While there are potential limitations with CRISPR‐CAS generation of knockouts, the fact that we obtained similar results with two completely different CLPP knockout lines, strongly supports the specificity of the compound effects on ClpP. As with any animal study, there are also limitations to our animal studies. We performed our analysis with the least number of animals to achieve a statistically powered result. Given the fact that ONC201 has been extensively examined in other animal models, we only tested the effects of the TR compounds in these studies. While female mice were used exclusively in our experiments, we acknowledge that a similar study in male mice could be valuable. However, since the focus of our study was breast cancer, we believe that our rationale for this initial study was logical. Last, we acknowledge the limitations of studying a single xenograft model. Ideally, additional studies will be performed in syngeneic models of breast cancer to fully characterize these important new compounds.

## AUTHOR CONTRIBUTIONS

Participated in research design: E. Fennell, L.M. Graves, E.J. Iwanowicz, D. Karanewsky, P.R. Graves, E. Holmuhamedov, and A.A. Iwanowicz. Conducted Experiments: E. Fennell, J. Wynn, K. Drizyte‐Miller, E. Leung, and Y. Greer. Performed data analysis: E. Fennell, K. Drizyte‐Miller, E. Leung, Y. Greer, and D. Karanewsky. Contributed new reagents or analytic tools: L. Aponte‐Collazo, Y. Greer, S. Lipkowitz, E.J. Iwanowicz, H. Lang, and H. Ashamalla. Wrote or contributed to the writing of the manuscript: E. Fennell, L.M. Graves, C. Der, S. Lipkowitz, and W. Houry.

## FUNDING INFORMATION

This research was supported by the National Institutes of Health National Institute of General Medical Sciences [Grant 5R01GM138520‐02]; National Institutes of Health National Cancer Institute [Grants R35CA232113, T32CA009156, ZIA SC 007263]; Canadian Institutes of Health [Grant PJT‐173345]; and the Canadian Cancer Society Innovation Grant [Grant 706282].

## DISCLOSURES

CJD is a consultant/advisory board member for Anchiano Therapeutics, Boragen, Day One Biotherapeutics, Deciphera Pharmaceuticals, Mirati Therapeutics, Revolution Medicines, SHY Therapeutics, and Verastem Oncology. CJD has received research funding support from Boragen, Deciphera Pharmaceuticals, Mirati Therapeutics, and SpringWorks Therapeutics and has consulted for Eli Lilly, Jazz Therapeutics, Ribometrix, Sanofi, and Turning Point Therapeutics. EJI and HL both have a financial interest in Madera Therapeutics.

## ETHICS STATEMENT

All animal experiments were performed in accordance with ethical and human guidelines. CR Discovery Services complies with the recommendations of the Guide for Care and Use of Laboratory Animals with respect to restraint, husbandry, surgical procedures, feed and fluid regulation, and veterinary care. The animal care and use program at CR Discovery Services is accredited by the Association for Assessment and Accreditation of Laboratory Animal Care International.

## Supporting information


Appendix S1

## Data Availability

Raw data can be made available upon request from the authors.
