# Supplementary material for: Characterization of TR‐107, a novel chemical activator of the human mitochondrial protease ClpP
Source: Pharmacol Res Perspect. 2022 Aug 5;10(4):e00993. doi: 10.1002/prp2.993 (PMC9354705; doi:10.1002/prp2.993)
Supplement: Supplementary file 1 — Appendix S1 [file PRP2-10-e00993-s001.docx]

Supplemental Data

Characterization of TR-107, a Novel Chemical Activator of the Human Mitochondrial Protease ClpP

Emily M. J. Fennell^1^, Lucas J. Aponte-Collazo^1^, Joshua D. Wynn^1^, Kristina Drizyte-Miller^1^, Elisa Leung^2^, Yoshimi Endo Greer^3^, Paul R. Graves^4^, Andrew A. Iwanowicz^5^, Hani Ashamalla^4^, Ekhson Holmuhamedov^6^, Henk Lang^5^, Donald S. Karanewsky^5^, Channing J. Der^1^, Walid A. Houry^2,7^, Stanley Lipkowitz^3^, Edwin J. Iwanowicz^5^ and Lee M. Graves^1*^

^1^Department of Pharmacology and the Lineberger Comprehensive Cancer Center, University of North Carolina at Chapel Hill, Chapel Hill, North Carolina. ^2^Department of Biochemistry, University of Toronto, Toronto, Ontario M5G 1M1, Canada. ^3^Women’s Malignancies Branch, Center for Cancer Research, National Cancer Institute, National Institutes of Health, Bethesda, Maryland. ^4^New York Presbyterian Brooklyn Methodist Hospital, Department of Radiation Oncology, Brooklyn, New York. ^5^Madera Therapeutics LLC, Chapel Hill, North Carolina. ^6^Institute of Theoretical and Experimental Biophysics, Russian Academy of Sciences, Pushchino, Russian Federation, 142292. ^7^Department of Chemistry, University of Toronto, Toronto, Ontario M5S 3H6, Canada.

**Corresponding Author**: Lee M. Graves, Department of Pharmacology, University of North Carolina, Chapel Hill, NC 27599. E-mail: lmg@med.unc.edu

Supplemental Figures and Figure Legends


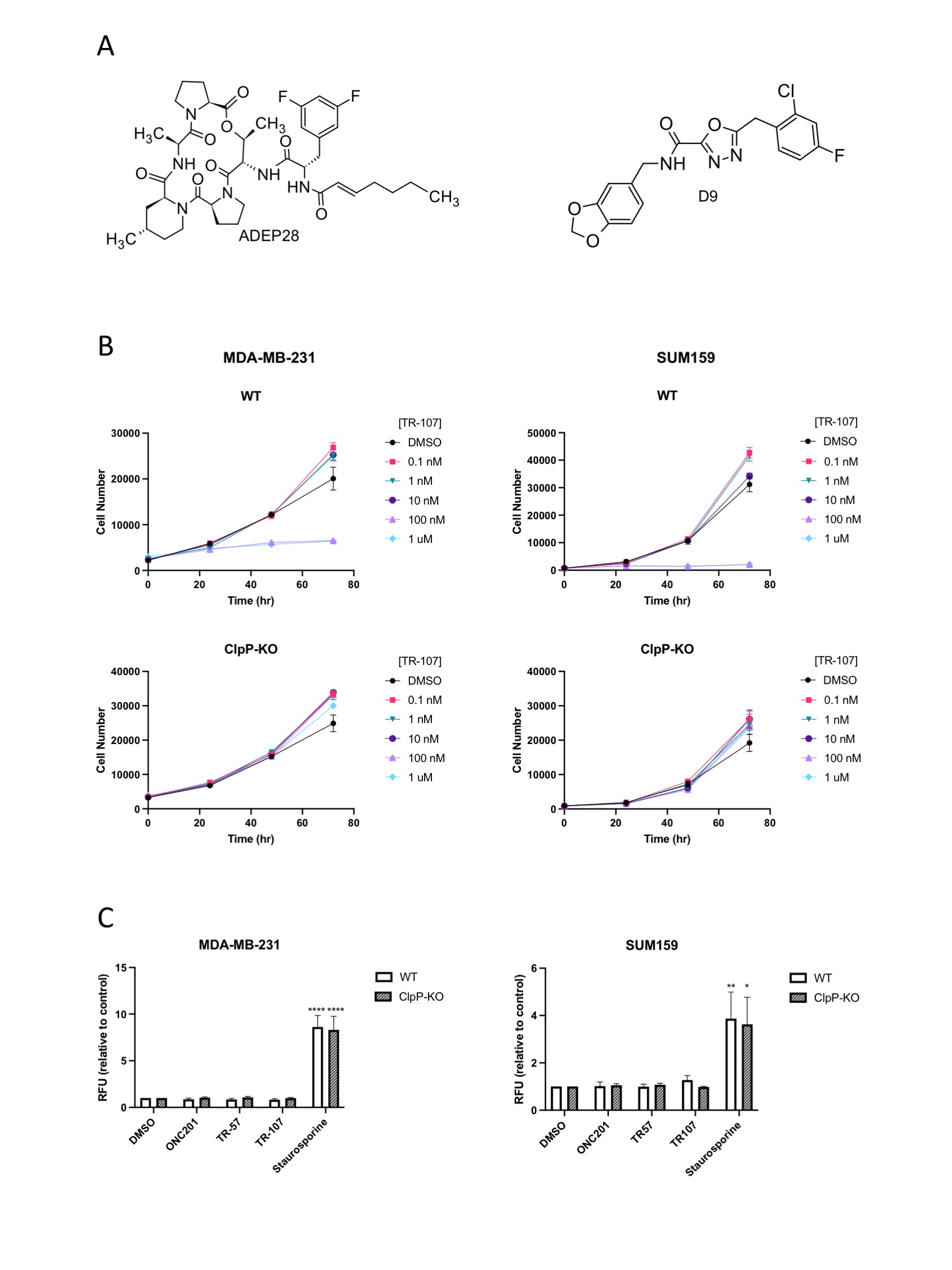


**Figure S1**. **TR compounds do not induce cell death in breast cancer cell lines.** A) Chemical structures of ClpP activators ADEP28 and D9. B) Total cell count assay in SUM159 and MDA-MB-231 cells (WT and ClpP-KO). Cells were treated with indicated drug concentrations for 0, 24, 48, and 72 hours and counted using Hoechst stain. Values represent mean cell count ± SEM, Representative of N=2. C) *In vitro* caspase-3/7 activity assay in SUM159 and MDA-MB-231 cells (WT and ClpP-KO). Cells were treated with 10 µM ONC201, 150 nM TR-57, 100 nM TR-107 or 100 nM staurosporine for 24 hours and caspase activity was measured via fluorometric caspase activity assay. Values represent mean fluorescence value ± SEM, N=3, p-value < 0.05 (*), 0.01(**), 0.0001 (****)


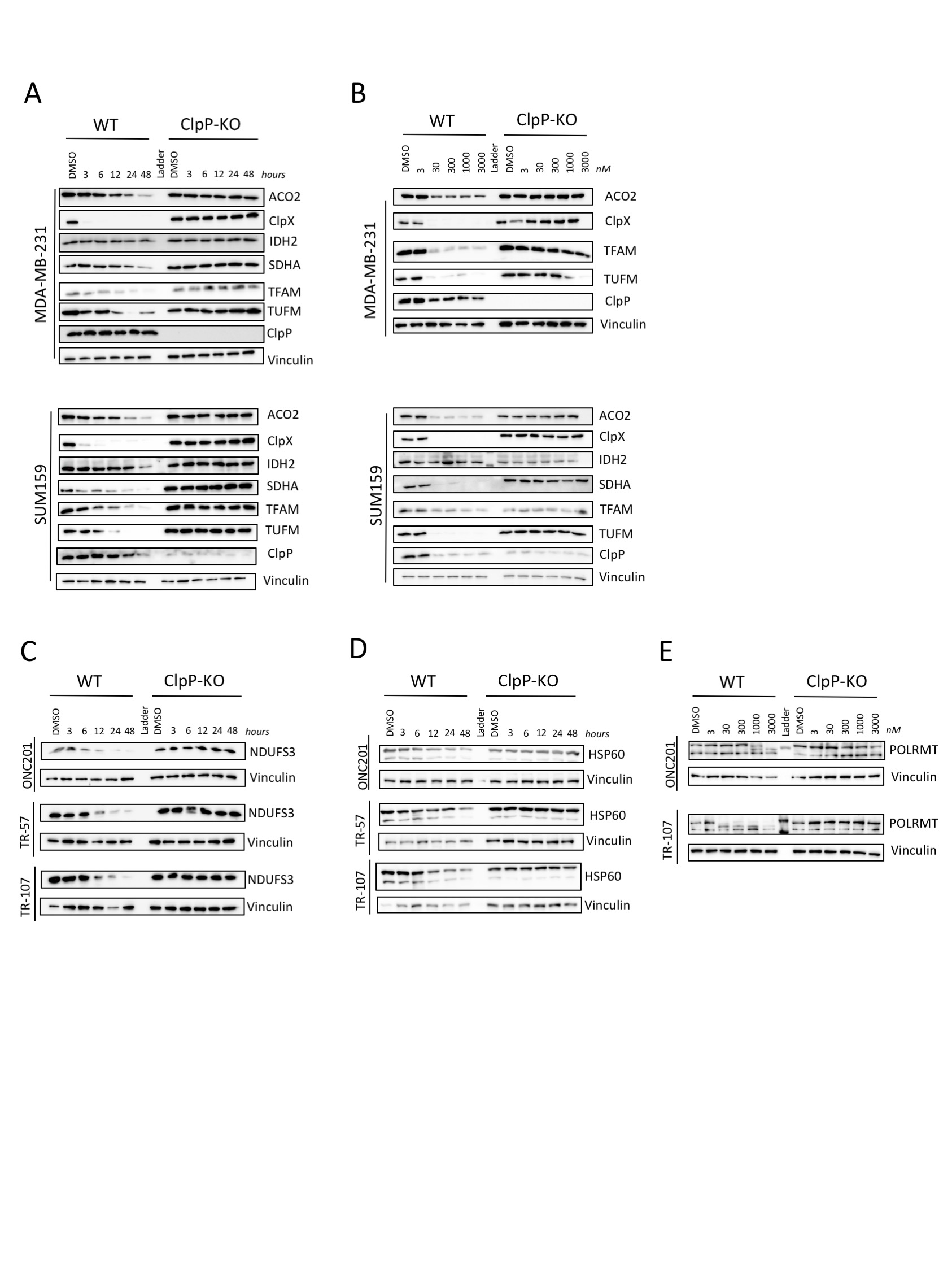


**Figure S2. ONC201, TR-107 and TR-57 induce loss of mitochondrial proteins in TNBC cells in a ClpP-dependent manner.** MDA-MB-231 and SUM159 cells were treated with 150 nM TR-57, for indicated timepoints (A) or with indicated doses for 24 hours (B) and immunoblot was performed for mitochondrial metabolic proteins. MDA-MB-231 cells were treated with 10 μM ONC201, 150 nM TR-57, or 100 nM TR-107 for indicated timepoints and immunoblotted for (C) NDUFS3 and (D) HSP60. (E) MDA-MB-231 cells treated with indicated concentrations of ONC201 or TR-107 for 24 hours were immunoblotted for POLRMT. N=3 (A, B) and N=2 (C-E).


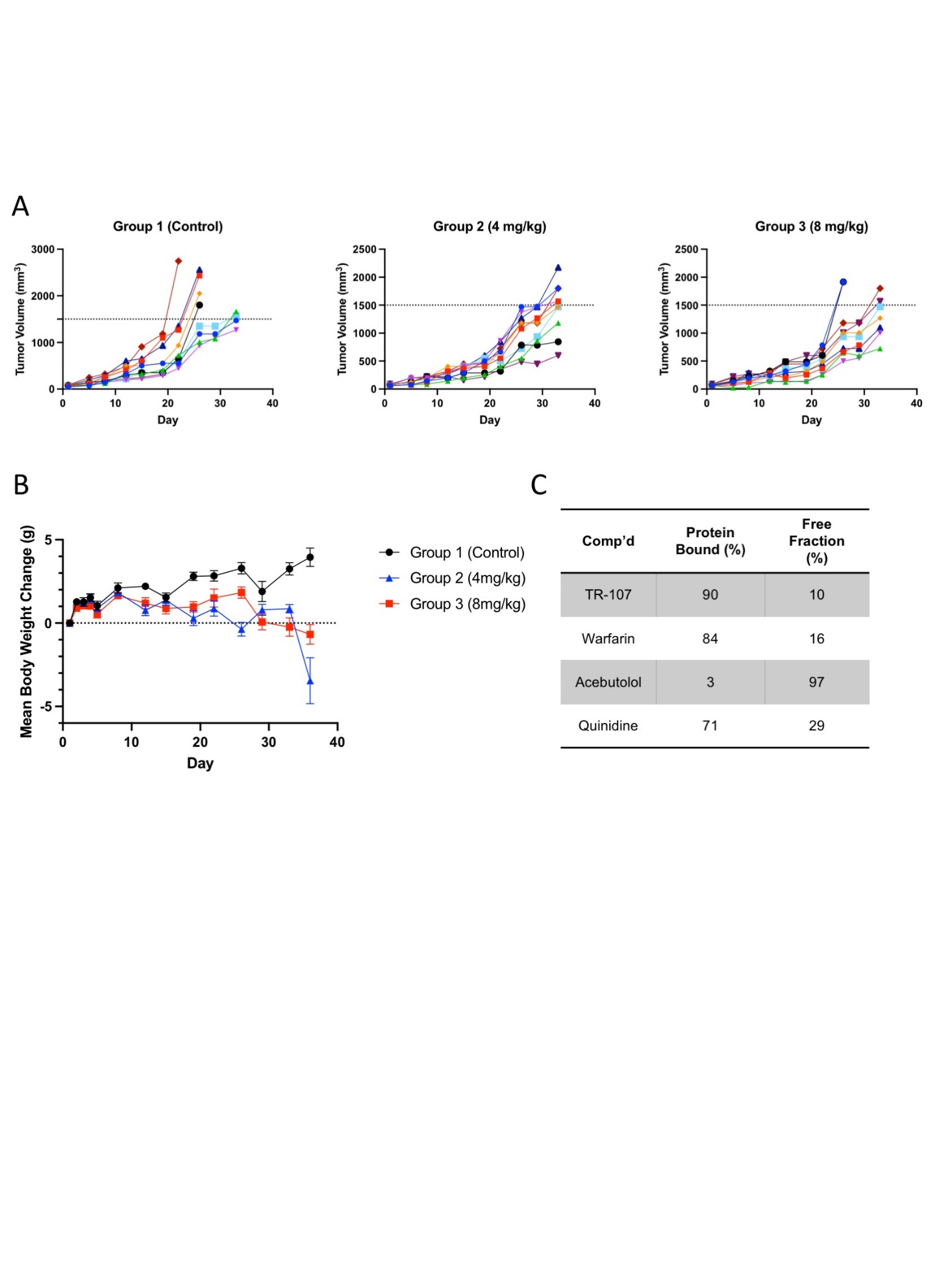


**Figure S3. TR-107 inhibits tumor growth in MDA-MB-231 mouse xenograft model.** A) Graphs of individual tumor volume measurements at indicated time points. Each color represents a single mouse participating in the study, N=10 per group. B) Average change in body weight compared to initial measurement of mice receiving vehicle (Group 1) or TR-107 treatment (Group 2 (4 mg/kg) or Group 3 (8 mg/kg)) at indicated time points. Values represent average ± SEM. N=10 per group. C) Murine plasma protein binding profile of TR-107 and control compounds (Warfarin, acebutolol, and quinidine). Values represent mean of N=2 replicates.

**Table S1. TR-107 dosing and treatment protocol for murine MDA-MB-231 xenograft study.** All compounds were dissolved in vehicle as described in Methods and were administered orally (P.O.) to 10 mice per group on schedules indicated.

**
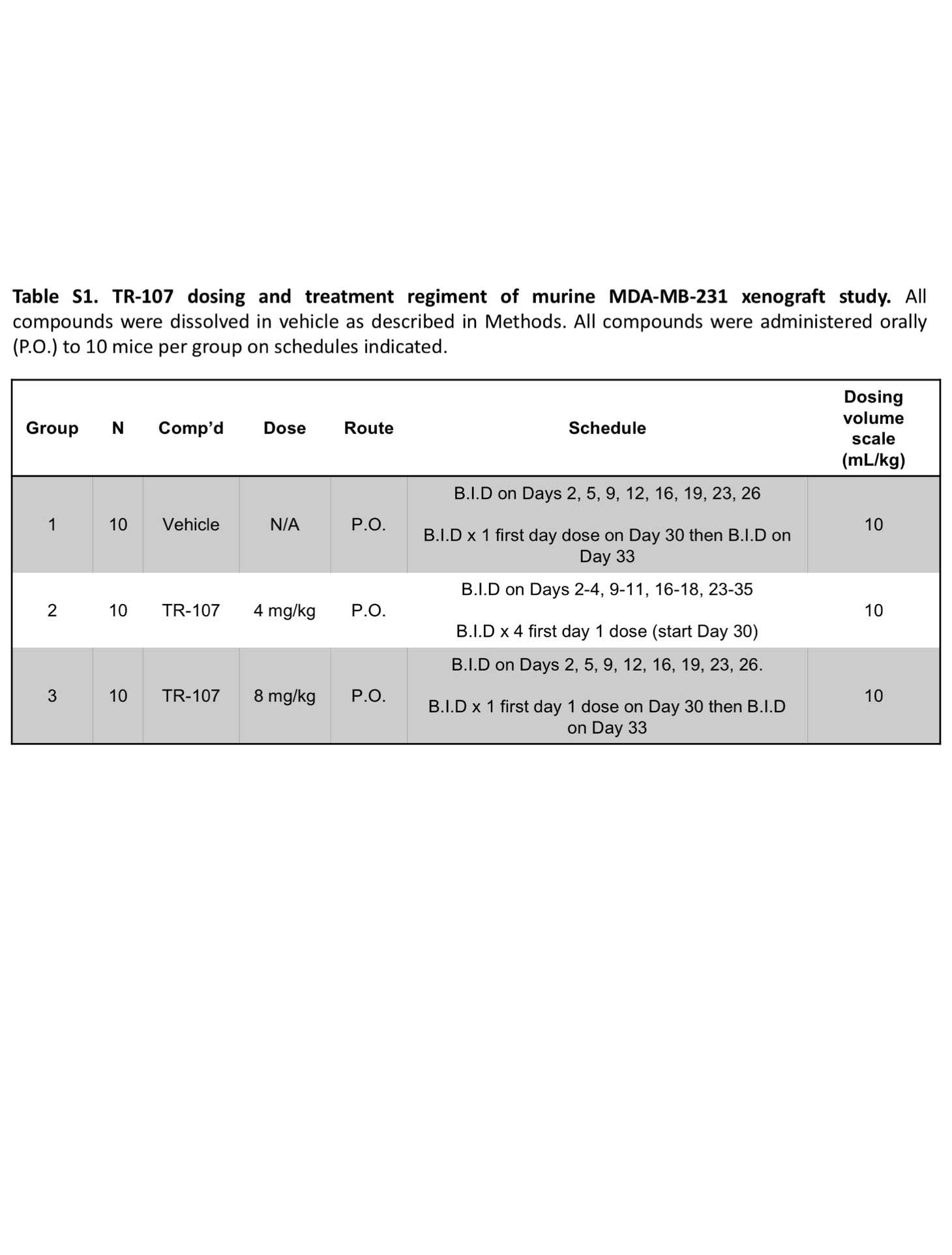
**

Supplemental Methods

**Immunoblotting**

Primary and secondary antibodies used in immunoblot analysis are listed below:

| Name | Manufacturer | Catalog Number |
| --- | --- | --- |
| Anti-SDHA | Cell Signaling Technologies | 5839 |
| Anti-ClpP |  | 14181 |
| Anti-ACO2 |  | 6922 |
| Anti-TFAM |  | 8076 |
| Anti-HMGSC2 |  | 36877 |
| Anti-TUFM | Invitrogen | PA5-27511 |
| Anti-IDH2 |  | PA5-79436 |
| Anti-POLRMT |  | PA5-28196 |
| Anti-ClpX | Abcam | 168338 |
| Anti-NDUFS3 |  | 183733 |
| Anti-HSP60 | BD Transduction Laboratories | 611562 |
| Anti-PYCR2 | Sigma-Aldrich | HPA056873 |
| Anti-mtTFA (TFAM) | Santa Cruz Biotechnologies | sc-376672 |
| Anti-Vinculin |  | sc-73614 |
| Anti-HSC70 |  | sc-7298 |
| Anti-Rabbit IgG HRP conjugate | Promega | W401B |
| Anti-Mouse IgG HRP conjugate |  | W402B |
